# *Enterobacter cloacae*: a villain in CaOx stone disease?

**DOI:** 10.1007/s00240-022-01311-8

**Published:** 2022-02-06

**Authors:** Yuanyuan Yang, Senyuan Hong, Jinzhou Xu, Cong Li, Shaogang Wang, Yang Xun

**Affiliations:** Department of Urology, Tongji Hospital, Tongji Medical College, Huazhong University of Science and Technology, Wuhan, 430030 Hubei China

**Keywords:** Kidney stone, Microbiome, *Enterobacter cloacae*, Flagellin

## Abstract

To explore the roles microbiome of urinary tract played in calcium oxalate stones (CaOx) formation, we collected two sides’ pelvis urine of patients with unilateral CaOx stones to set self-control to diminish the influence of systemic factors. Patients with unilateral CaOx stones were recruited in our study according to strict criteria. 16S rRNA gene sequencing was applied to every pair of pelvis urine. Bacterial genome sequencing of *Enterobacter cloacae* was conducted and bioinformatic analysis was applied to explore the possible pathways of *Enterobacter cloacae* inducing CaOx stones formation. In vivo experiments were conducted to validate our claims. Von Kossa staining, TUNEL assay and Western Blot were applied to SD rats exploring the mechanism of stone formation. We found 26 significantly different bacteria between stone sides and non-stone sides’ pelvis urine, among which *Enterobacter cloacae* ranked the most different. Bacterial genome sequencing of *Enterobacter cloacae* revealed that its virulence factors included Flagellin, LPS and Fimbrial. GO and KEGG analysis revealed it probably induced CaOx stone formation via ion binging and signaling transduction pathways. The results of animal experiments indicated that Glyoxylic Acid could promote apoptosis and crystal depositions of kidney comparing with control group while pre-injected with *Enterobacter cloacae* could apparently compound the effects. While Western Blot demonstrated that Glyoxylic Acid or *Enterobacter cloacae* could increase the expression of IL-6, Mcp-1, BMP2 and OPN in rats’ kidney, Glyoxylic Acid and *Enterobacter cloacae* together could aggravate these increases. These findings indicated that *Enterobacter cloacae* might play important roles in CaOx stones formation. However, this study is just a preliminary exploration; further studies still need to be conducted.

**Supplementary Information:**

The online version contains supplementary material available at 10.1007/s00240-022-01311-8.

## Introduction

Kidney stone is a common disease with substantial morbidity and high recurrence rate. The prevalence and recurrence rate of stone formation is on the rise worldwide [1]. In China, the incidence of kidney stones is estimated to be 5.8%, including 6.5% in men and 5.1% in women [2]. Recurrence of kidney stones can cause severe damage, even renal failure which imposes heavy financial and healthy burden on individuals and society [3]. Recently, instrument miniaturization has greatly improved the surgical treatment of kidney stones. However, medical therapies and prevention have not improved substantially because the mechanism of stone formation remains unclear [4]. About 80% of kidney stones are composed of CaOx crystals mixed with varying amounts of calcium phosphate (CaP) [5]. High urinary oxalate is considered to be a crucial risk factor [6], while the urinary calcium is often normal or transient high [7]. So far, how CaOx stones form still remains unclear. Previous studies have focused on systematic factors, such as metabolic or genetic problems [8]. However, both our clinical observation and literature reports indicate that most patients with CaOx stones have unilateral nidus [9]. And all of these can’t be explained by systematic factors. Hence, there may be some local factors in the kidney inducing the formation of CaOx stones.

Historically, it is widely acknowledged that urinary microbiota is closely related to the formation of infectious stones, bacteria can promote the formation of struvite stones via urease [10]. Recently, with the rapid development of 16S rRNA gene sequencing, detection of the microbiome has been widely improved [11]. Thus, increasing evidence shows that bacteria are more broadly associated with kidney stones, not only infectious stones, but also CaOx stones [12, 13]. Ryan et al. isolated and purified *Escherichia coli* and *Staphylococcus epidermidis* from urine and stones of patients with CaOx stones, suggesting that the formation of calcium oxalate stone may be related to urinary microbiota [14]. However, the urine samples in this study were collected from bladder of different patients, which cannot eliminate the influence of systemic factors, such as genetics and metabolism factors.

To eliminate the influence of metabolic and genetic abnormalities, we recruited patients with unilateral CaOx stones and collected their two sides (both the stone sides and the non-stone sides) pelvis urine. We applied 16S rRNA gene sequencing to detect the differences of microbiota between the stone sides and non-stone sides pelvis urine of the same patients. We also performed an in vivo experiment to preliminarily explore the relationship between microbiota and CaOx stones. This study, to our knowledge, registers the first attempt to disclose the different microbiota of renal pelvis urine between stone side and non-stone side of a same patient, trying to reveal the relationship between urinary microbiota and CaOx stones.

## Methods

### Participants’ information and urine samples collection

Patients came to our department suspected unilateral CaOx stones which predicted by Computed Tomography were invited to participate in the study. Exclusion criteria included antibiotic exposure prior to 4 weeks, hematuria or other organic diseases. All patients received percutaneous nephro-lithotomy. Pelvis urine derived from each participant were collected at time of percutaneous nephro-lithotomy, after intravenous injection of furosemide, renal pelvis urine was directly collected in an asepsis tube through a ureteral catheter. We collected pelvis urine of both stone sides and non-stone sides. Samples were immediately frozen in liquid nitrogen, and subsequently stored under – 80 ℃. The whole process maintained sterility via the instruction of MICROCOSM (an international consortium for microbiome in urinary stone disease) [15]. After stone composition analysis, we selected urine samples of patients with a definite diagnosis of CaOx stones and applied 16S rRNA gene sequencing. The samples processing and data analysis section were also followed the consortium’s instructions to minimize the technical biases and barriers associated with microbiome research. Ethical Review Board of Tongji Hospital, Tongji Medical College, Huazhong University of Science and Technology has approved the collection of renal pelvis urine samples (2021S130). Informed consents were obtained from each participant.

### Separation and purification

*Enterobacter cloacae* isolates were collected from pelvis urine of patients recruited in our study using *Enterobacter Cloacae* Isolation Agar (SoleyBio, Beijing, QP0382). Whole Genome Sequencing (WGS) was used to confirm the *Enterobacter cloacae* and explore the characteristics of it.

### 16S rRNA gene sequencing and Whole Genome Sequencing (WGS)

Total genomic DNAs were extracted using the OMEGA Soil DNA Kit (D5625-01) (Omega Bio-Tek, Norcross, GA, USA) and stored at − 20 °C prior to further analysis. PCR amplification of the nearly full-length bacterial 16S rRNA genes was performed using the forward primer 27F (5′-AGAGTTTGA TCMTGGCTCAG-3′) and the reverse primer 1492R (5′-ACCTTGTTACGACTT-3′). The extracted DNA’s amplification was conducted via two-step PCR, with sample-specific 16-bp barcodes incorporated into the forward and reverse primers in the second PCR step. After quantification, amplicons were pooled in equal amounts, and Single Molecule Real-Time (SMRT) sequencing was performed using the PacBio Sequel platform. Raw sequences were elaborated through the PacBio SMRT Link portal (version 5.0.1.9585). Microbiome bioinformatics were performed with QIIME2 (https://docs.qiime2.org/2019.4/tutorials/) [16]. Raw sequence data were de-multiplexed and quality-filtered, de-noised, merged and chimera removed using the DADA2 plugin [17]. Taxonomy was assigned to amplicon sequence variants (ASVs) using the classify-sklearn naïve Bayes taxonomy classifier in feature-classifier plugin against the SILVA Release 132 Database [18, 19].

### Bioinformatics and statistical analysis

Sequence analysis was mainly performed using QIIME2 and R packages (v3.2.0). Alpha diversity indices, such as Chao1, Simpson index, Shannon index, Pielou’s evenness, Observed species, Faith’s PD and Good’s coverage, were calculated using the ASV table in QIIME2, and visualized as box plots. Beta diversity analysis was conducted to explore the structural variation of microbial communities across Hea and Lith groups using unweighted uniFrac distance metrics [20], visualized as principal coordinate analysis (PCoA) and evaluated by the Adonis test [21]. Venn diagram was generated to visualize the common and unique ASVs among Hea groups and Lith groups via R package “Venn Diagram” [22]. Linear discriminant analysis Effect Size (LEfSe) analysis was used to identify differentially abundant bacteria among the Hea groups and Lith groups with a cutoff of 2.0. PICRUSt2 package was used to conduct the Kyoto Encyclopedia of Genes and Genomes (KEGG) pathways analysis, Gene Ontology analysis and Metacyc pathways analysis.

### Animal experiment design

Twelve male SD rats (8 weeks old, 300 g) were purchased from the experimental Animal Centre of Tongji Hospital, Tongji Medical College, Huazhong University of Science and Technology. All procedures were approved by the Animal Care and Use Committee of Tongji Hospital, Tongji Medical College, Huazhong University of Science and Technology. Rats were acclimatized to the environment of 12 h light/dark cycle for 1 week in a specific pathogen-free animal house. Rats were randomly divided into the following four groups of 3 rats each: control group; Glyoxylic Acid group, intraperitoneally injected with Glyoxylic Acid (10.5 mg/ml, 6.66 ml/kg, MackLin, Shanghai) every day for nine days; *Enterobacter cloacae* group, renal pelvis injection of *Enterobacter cloacae* (2*10^8^ cfu/ml, 50 ul); Glyoxylic Acid + *Enterobacter cloacae* group, renal pelvis injection of *Enterobacter cloacae* (2*10^8^ cfu/ml, 50 ul), then intraperitoneally injected with Glyoxylic Acid (10.5 mg/ml, 6.66 ml/kg, MackLin, Shanghai) every day for nine days. All rats were euthanized after the above treatment for 2 weeks. The renal tissues were collected, with fixed with 4% paraformaldehyde or frozen at − 80 °C for further use.

### Detection of crystal deposition by Von Kossa staining and apoptosis by TUNEL Assay

Apoptoses of the kidney tissues were assessed with TUNEL assay, following the manufacturer’s instructions (Vazyme). Images were captured using a BX53 fluorescence microscope (Olympus, Tokyo, Japan). The crystal depositions in the kidneys were analyzed by Von Kossa staining according to manufacturer’s instructions (Solarbio, Beijing). The stained tissues were observed by microscopy (Olympus, Japan).

### Western blot analysis

The kidney tissues were cut up and lysed in RIPA Lysis Buffer with protease inhibitor phenylmethanesulfonyl fluoride (PMSF) and phospho-proteinase inhibitors (Beyotime Biotechnology, Shanghai, China). The protein concentration was measured using the bicinchoninic acid (BCA) protein assay kit (Beyotime Biotechnology, Shanghai, China). Proteins were separated and isolated using sodium dodecyl sulfate–polyacrylamide gel electrophoresis with 5% and 12% for 120 V, 2 h and then transferred onto polyvinylidene fluoride (PVDF) membranes for 200 mA, 70 min (MCP-1, IL-6) and 200 ma, 90 min (BMP2, OPN). The PVDF membranes were blocked with 5% bovine serum albumin for 2 h. The membranes were incubated with primary antibody against BMP2 (proteintech group, 66,383–1-Ig, China, 1:1500), OPN (Boster, PB0589, China, 1:1000), IL-6 (Boster, BA4399, China, 1:1000), MCP-1 (proteintech, 66,272–1-Ig, China, 1:1500), β-actin (proteintech, 66,009–1-Ig, China, 1:4000) at 4 °C overnight. Then they were incubated with secondary antibody at room temperature for 2 h, the proteins were visualized using enhanced developer (Boster, China). The gray values of these proteins were analyzed with image-pro plus. All the WB quantifications were used three independent Western blot results.

## Results

### General characteristics of patients with CaOx stones

Patients with unilateral CaOx stones were involved in this study. Finally, we brought 4 patients, that is to say, 8 samples into our study according to our strict inclusion criteria. Detailed information of participants was listed in Table 1. These kidney stones were primarily composed of calcium oxalate. The calcium concentration of these participants was normal. The 16S rRNA gene sequencing was applied to both the stone side and non-stone side pelvis urine of every participant to eliminate systemic factors like metabolic and genetic factors.

**Table 1:** Basic information of patients included in this study

ID	Age (y)	Gender	BMI (kg/m^2^)	Plasma Ca (mmol/L)	Stone side	Stone type
1	68	Female	23.8	2.31	Right	CaOx
2	42	Male	30	2.25	Left	CaOx
3	42	Male	27.9	2.33	Left	CaOx
4	60	Male	26.3	2.2	Right	CaOx

*CaOx* Calcium oxalate

### Richness and diversity of the pelvis urine microbiota in stone sides and non-stone sides

**Fig. 1:**
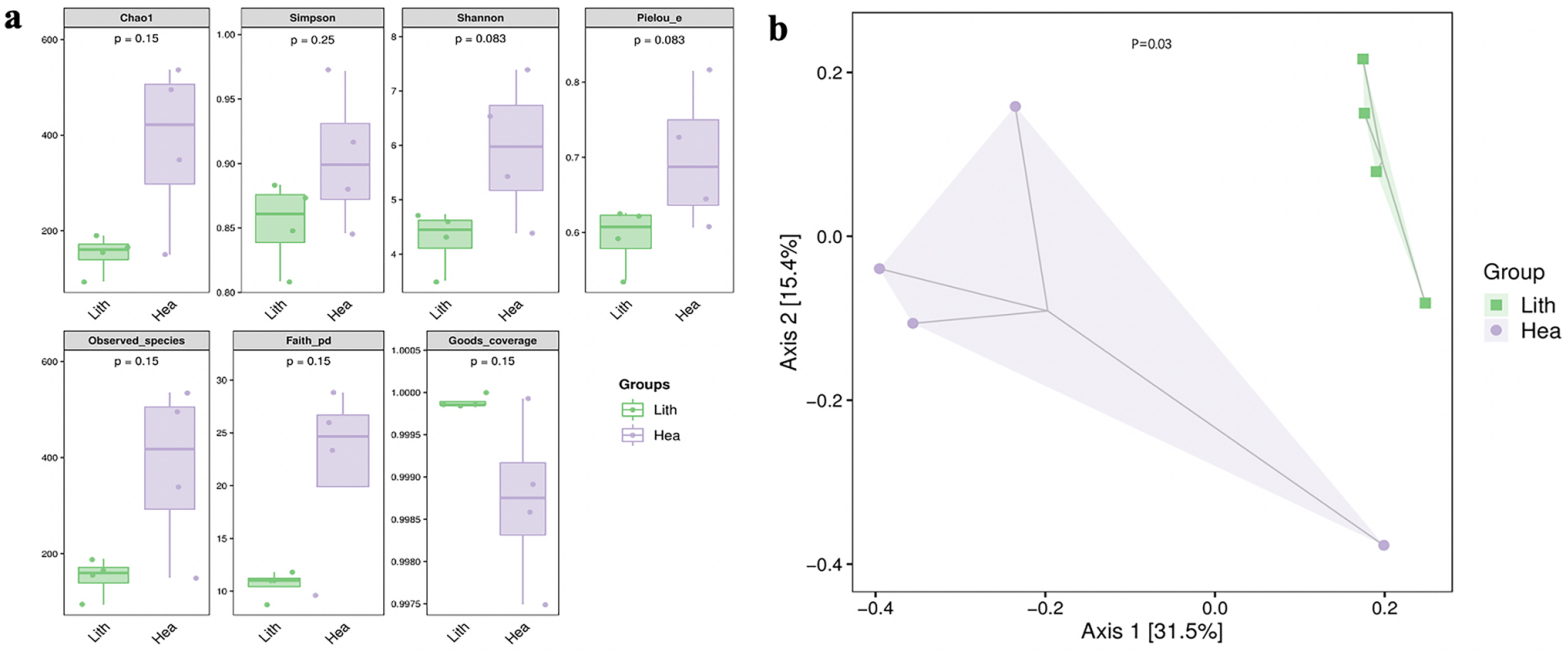
Richness and diversity of the pelvis urine microbiota in stone sides and non-stone sides. **a** Comparison of alpha diversity of pelvis urine microbiota among both non-stone sides (Hea) and stone sides (Lith). Chao1, Shannon, Simpson, Pielou_e, observed species, Faith_ pd and Goods coverage indices at amplicon sequence variants (ASVs) level were compared between both non-stone sides (Hea) and stone sides (Lith) by the Kruskal–Wallis test. **b** Comparison of beta diversity of pelvis urine microbiota among both non-stone sides (Hea) and stone sides (Lith). Beta diversity is conducted via unweighted unifrac-based PCoA, PCoA scatter plot based on binary Pearson distance at ASVs level revealed classification of non-stone sides and stone sides

The pelvis urine derived from both two sides of each participant was applied to 16S rRNA gene sequencing analysis. A total of 103,198 high-quality sequences were obtained from these 8 samples with an average length of 1462. All the sequences were clustered into 1027 ASVs, which belonged to 794 genera and 58 phyla. The Kruskal–Wallis test showed that alpha diversity indices including Chao1, Simpson, Shannon and Observed species indicated no significant difference in within-habitat diversity (Fig. 1a). PCoA at the ASV level showed that the overall microbiota composition of the Lith (stone side) and Hea (non-stone side) groups were different, In other words, between-habitats diversity was different, which was confirmed by the adonis test (*P* < 0.01) (Fig. 1b).

### Taxonomic analysis of microbiota composition between stone sides and non-stone sides of unilateral CaOx stone patients

**Fig. 2:**
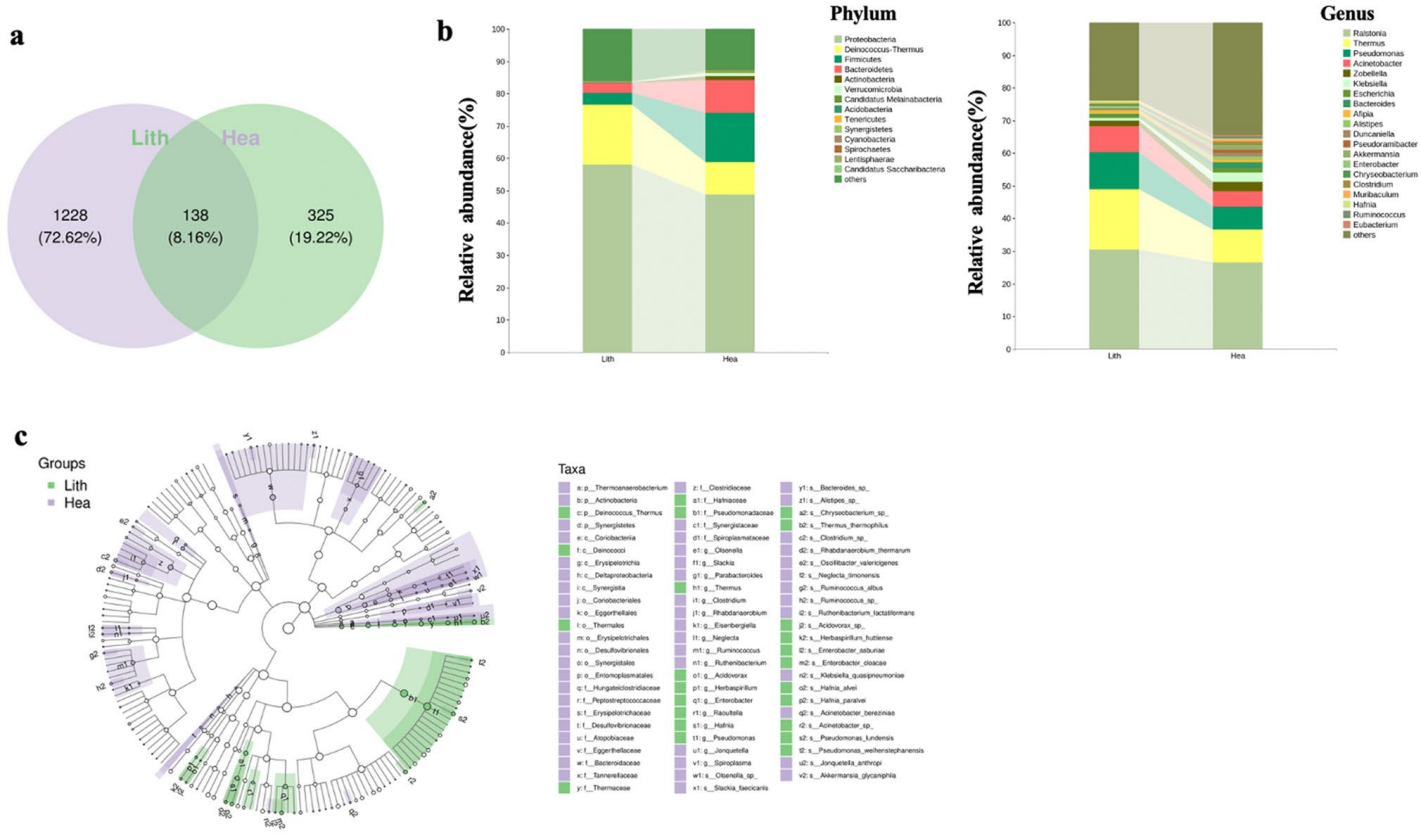
Taxonomic analysis of microbiota composition between stone sides and non-stone sides of unilateral CaOx stone patients. **a** A total of 1691 bacteria in pelvis urine were identified, of which 1228 were found in stone sides and 325 in non-stone sides. **b** The composition of the microbiota in Hea and Lith group at phylum or genus level. **c** The cladogram shows the classification hierarchy relationship of the main classification units from phylum to species (from inner to outer ring) in the sample community. The node size corresponds to the average relative abundance of the classification unit, and the hollow node represents a classification unit with no significant differences between groups, while solid nodes indicate significant intergroup

**Fig. 3:**
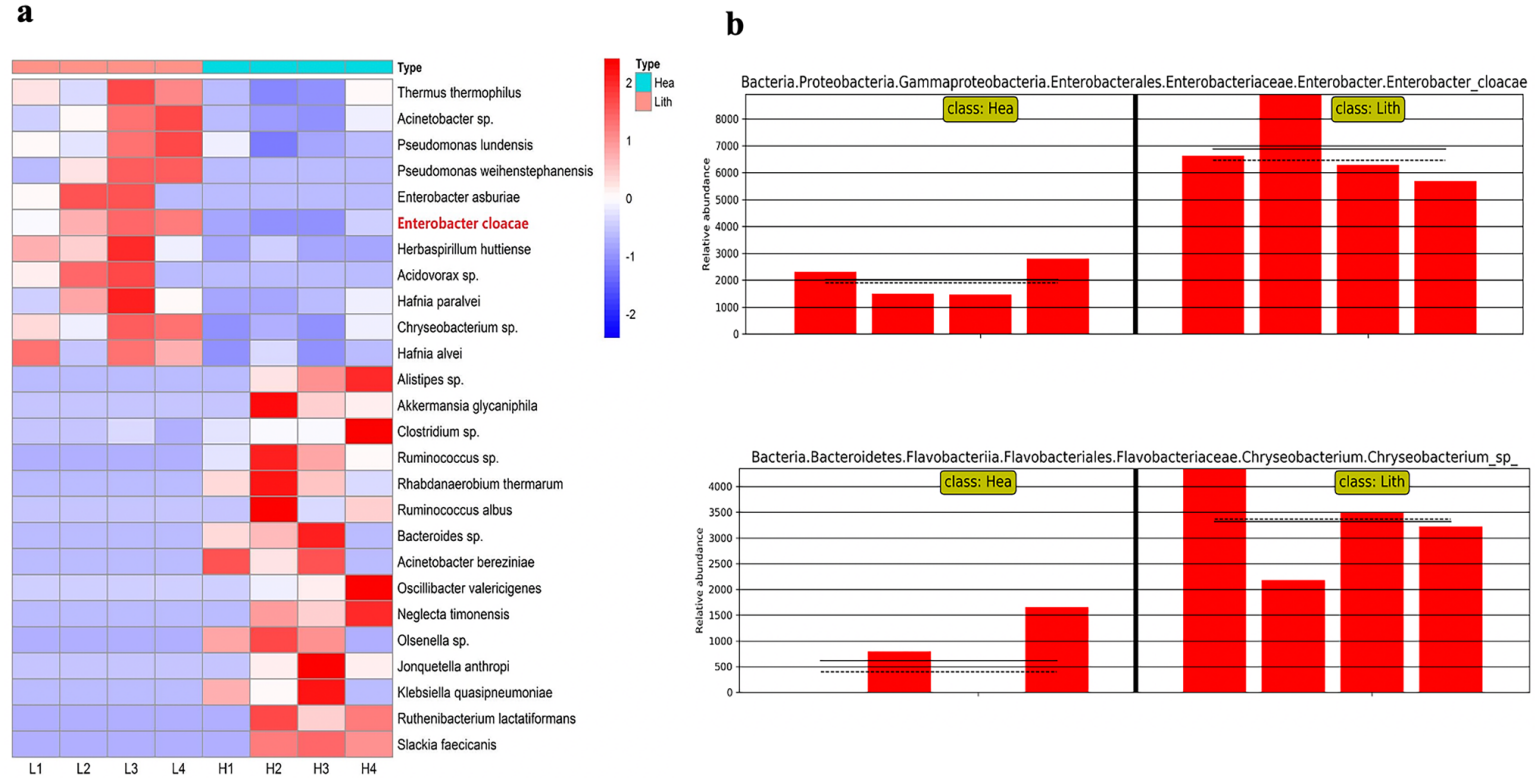
The different bacteria between non-stone sides (Hea) and stone sides (Lith). **a** With the screening criteria of *p* < 0.05, 26 species of bacteria were found to be most significantly different between two sides. The scale of red-blue spectrum represented the abundance after zero-mean normalization (*Z*-score). **b** The relative abundance of *Enterobacter cloacae* and *chryseobacterium *sp. in pelvis Urine of both stone sides (Lith) and non-stone sides (Hea). The scale of *y*-axis is the original relative abundance times 1,000,000 to include some very low abundance

Venn diagram showed that Lith and Hea groups shared only 138 (8.16%) ASVs in common, 1228 (72.62%) ASVs were identified only in Lith group and 325 (19.22%) only in Hea group (Fig. 2a). Taxonomic assignment of the ASVs revealed the composition of the bacterial population down to the phylum and genus level. At the phylum level, Proteobacteria was the most common bacteria in Lith groups, followed by Firmicutes. At the genus level, the common bacteria of Lith groups were *Pseudomonas*, *Acinetobacter*, *Klebsiella* and *Escherichia* (Fig. 2b). LDA effect size analysis (LEfSe) showed the classification hierarchy relationship of the main classification units from phylum to species (from inner to outer ring) in the sample community. From this cladogram, we found that 11 bacteria were stably rich in Lith groups at species level, which might play important roles in CaOx stones (Fig. 2c). To further compare the differences in species composition among samples and display the distribution trend of species abundance in each sample, we used a heatmap for species composition analysis. According to the screening bacteria (*P* < 0.05), we identified 26 most significantly different bacteria at species level. Detailed information of these 26 bacteria was shown in Table 2*. *Among these dysregulated bacteria, *Enterobacter cloacae* and *Chryseobacterium *sp. were the most stably up-regulated bacteria in stone side of unilateral CaOx stone patients’ pelvis urine (Fig. 3a). We could know the specific abundance of *Enterobacter cloacae* and *Chryseobacterium *sp. of each sample via the histogram of different groups (Fig. 3b).

**Table 2:** Basic information of 26 significantly different bacteria

Taxa (species)	Abundance	LDA score	*P* value	Gram staining	Group	Characteristic description
*Thermus thermophilus*	5.264	4.610	0.021	Negative	Stone	PHA synthesis
*Acinetobacter* sp.	4.281	3.76	0.021	Negative	Stone	Biodegradation Bioremediation
*Pseudomonaslundensis*	4.250	3.706	0.021	Negative	Stone	Hospital Infection
*Pseudomonas weihenstephanensis*	2.845	3.559	0.047	Negative	Stone	Hospital Infection
*Enterobacter asburiae*	2.743	3.459	0.047	Negative	Stone	Aid plant growth Degrade polyethylene
*Enterobacter cloacae*	3.838	3.459	0.021	Negative	Stone	Catalase Positive
*Herbaspirillum huttiense*	2.967	3.415	0.038	Negative	Stone	Bacteremia/RTI
*Acidovorax* sp.	2.947	3.322	0.047	Negative	Stone	Degrade Thiobencarb
*Hafnia paralvei*	3.644	3.290	0.043	Negative	Stone	Gastroenteritis
*Chryseobacterium* sp.	3.521	3.219	0.020	Negative	Stone	Degrade DDT
*Hafnia alvei*	3.390	3.109	0.042	Negative	Stone	Urosepsis\carcinoma of colon and rectum
*Alistipes* sp.	3.916	3.707	0.047	Negative	Control	Ulcerative Colitis
*Akkermansia glycaniphila*	3.987	3.702	0.047	Negative	Control	Degrade Mucoprotein
*Clostridium* sp.	3.883	3.534	0.021	Positive	Control	Gut microbiota
*Ruminococcus* sp.	3.677	3.419	0.014	Positive	Control	Association with ASD
*Rhabdanaerobium thermarum*	3.489	3.292	0.014	Positive	control	Isolated from a hot spring
*Ruminococcus albus*	3.301	3.248	0.047	Positive	control	Association with ASD
*Bacteroides* sp.	3.464	3.232	0.047	Negative	control	Opportunistic Infection
*Acinetobacter bereziniae*	3.213	3.173	0.047	Negative	control	Opportunistic Infection
*Oscillibacter valericigenes*	3.400	3.158	0.047	Positive	control	Isolated from the alimentary canal of a Japanese corbicula clam
*Neglecta timonensis*	2.814	3.138	0.047	Positive	control	Type 2 Diabetes
*Olsenella *sp*.*	3.072	3.125	0.047	Negative	control	Gut microbiota
*Jonquetella anthropi*	2.471	3.119	0.047	Negative	control	Oxidase- and catalase-negative
*Klebsiella quasipneumoniae*	3.217	3.080	0.047	Negative	control	bacterial hepatic abscess
*Ruthenibacterium lactatiformans*	2.991	3.074	0.047	Negative	control	Induce CD8 + T
*Slackia faecicanis*	2.800	3.063	0.047	Positive	control	catalase- and oxidase-negative

*PHA* Polyhydroxylalkanoate, *RTI* Respiratory tract infection, *ASD* Autistic Spectrum Disorder

### Function of pelvis urine microbiota

**Fig. 4:**
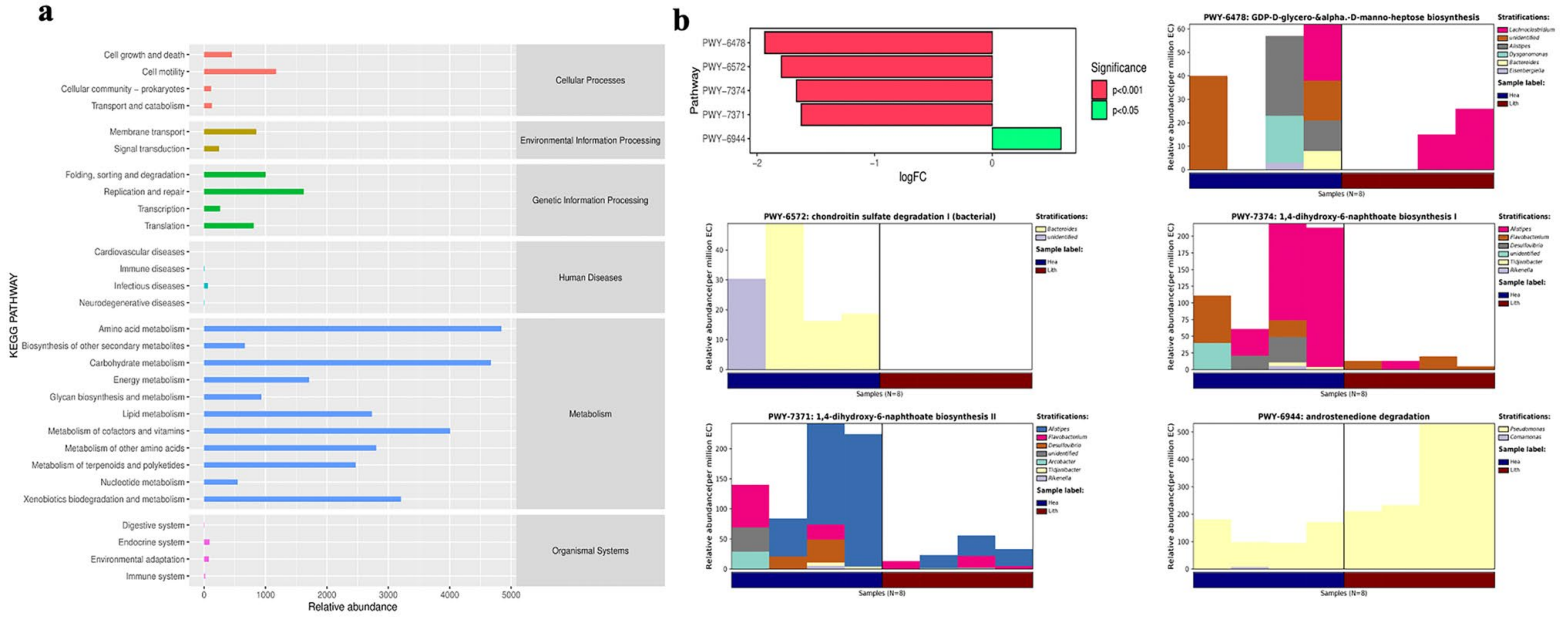
Bioinformatics analysis of microbiota of pelvis urine. **a** KEGG pathways analysis of pelvis Urine of both stone sides and non-stone sides. The unit of the *x*-axis is KO per 1,000,000. **b** The different MetaCyc pathways between stone sides and non-stone sides pelvis Urine of patients with CaOx stones

To explore the possible biological functions of bacteria derived from pelvis urine samples, we conducted KEGG pathway analysis (Fig. 4a). The results demonstrated that the most enriched pathways were related to cellular processes (cell motility), Environment Information Processing (Membrane transport and Signal transduction), Genetic Information Processing (Replication and Repair), Human diseases (Infectious diseases), Metabolism and Organismal Systems (Environmental adaptation and Immune system). MetaCyc pathways indicated that androstenedione degradation enriched in Lith groups (stone sides) and the primary dysregulated bacteria is *Pseudomonas* (Fig. 4b).

### Whole Genome Sequencing (WGS) of *Enterobacter cloacae* isolated and purified from pelvis urine

**Fig. 5:**
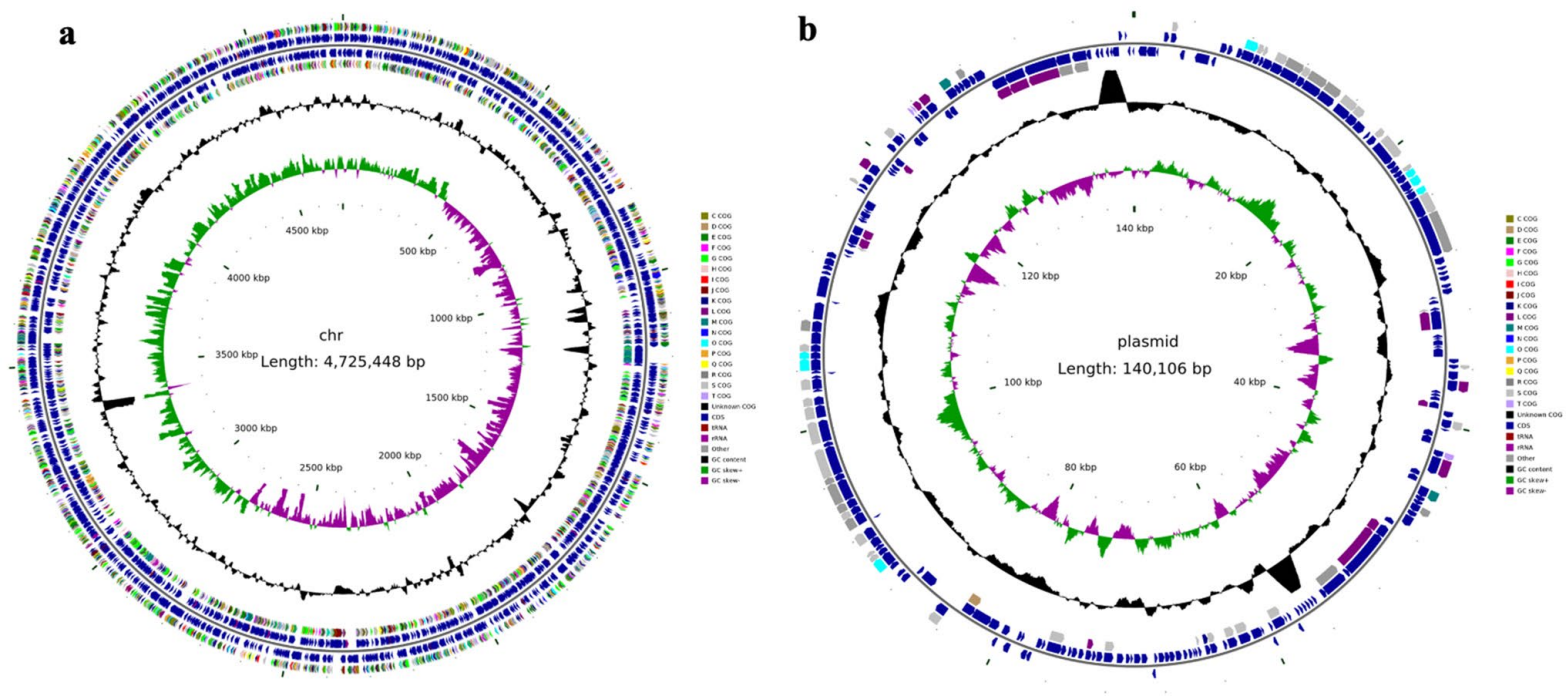
Genomic Graph of *Enterobacter cloacae*. **a** Genomic Graph of chromosome. **b** Genomic Graph of plasmid. From inside to outside, the first circle represents the scale; the second circle represents GC Skew; the third circle represents GC content; the fourth and seventh circles represent the COG to which each CDS belongs; the fifth and sixth loops represent the positions of CDS, tRNA, and rRNA on the genome

**Fig. 6:**
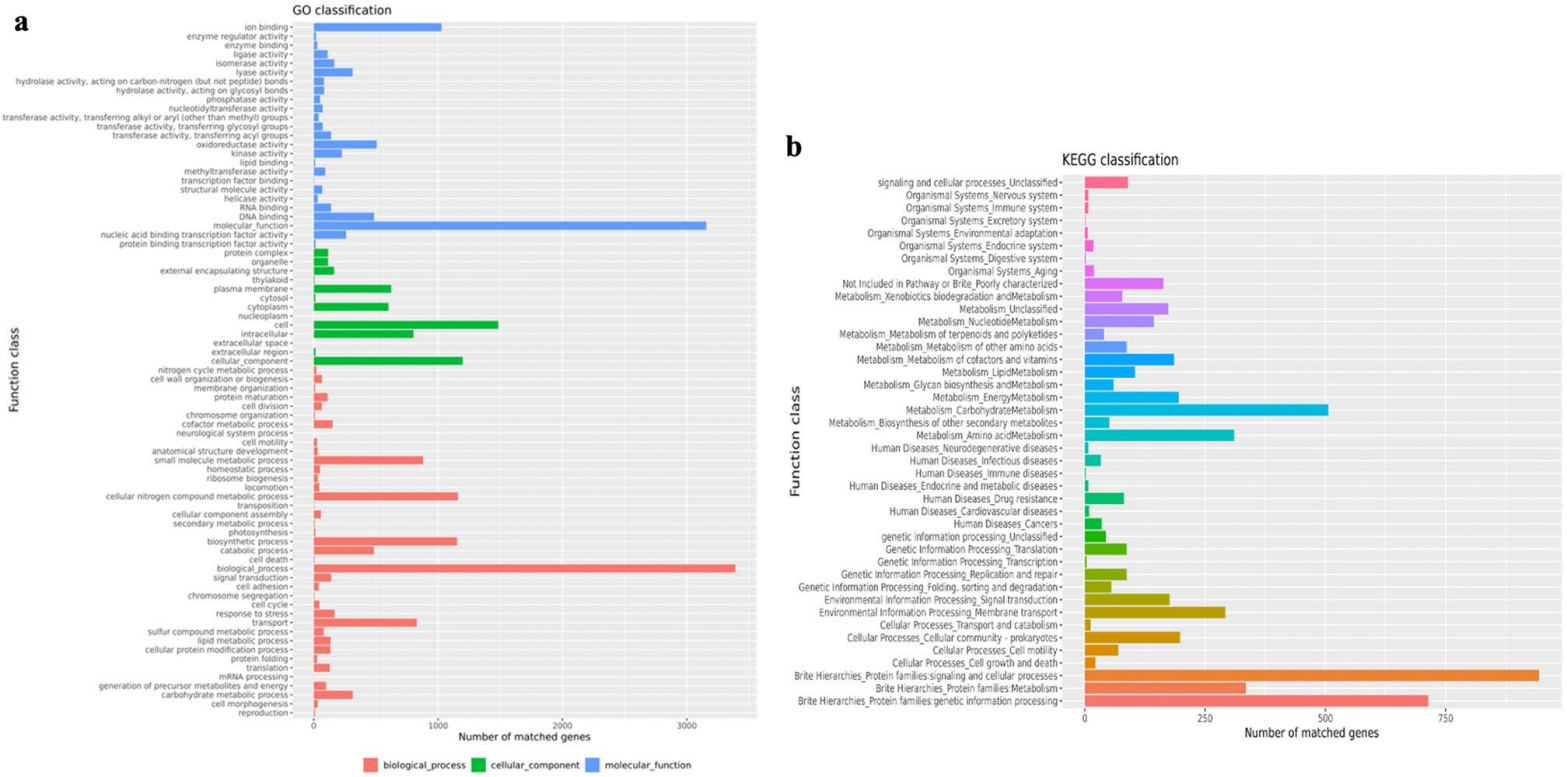
Bioinformatics analysis of *Enterobacter cloacae*. **a** Gene oncology analysis of *Enterobacter cloacae*. **b** KEGG pathways analysis of *Enterobacter cloacae*

To further explore *Enterobacter cloacae*, we conducted Whole Genome Sequencing of *Enterobacter cloacae* isolated and purified from pelvis urine. Genomic Graph made a comprehensive view of the *Enterobacter cloacae*, including plasmid and chromatin (Fig. 5). Furthermore, we performed GO and KEGG analysis to find possible pathways that *Enterobacter cloacae* utilized to promote stones formation. Bioinformatics results suggested that ion binding and signal transduction pathways are enriched. Given that CaOx stones are closely associated with calcium ions and signal transduction, *Enterobacter cloacae* may be a possible breakthrough for CaOx stone formation (Fig. 6). What’s more, we explored the possible virulence factors of *Enterobacter cloacae* and found proteins associated with flagellum were the primary factors (Table 3).

**Table 3:** Virulence factors of *Enterobacter cloacae*

VF-Name	Symbols VF	VF-Full Name
Flh	FlhA,FlhB,FlhC,FlhD	Flagellar biosynthesis protein
Che	cheA,cheB,cheW,cheY,cheZ	Chemotaxis protein
Fli	fliA,fliF,fliG,fliH,flil,fliN,fliP,fliQ,fliR,fliS	Flagellar biosynthetic protein
mot	motA,motB	Flagellar motor protein
flg	flgB,flgC,flaD,flaE,flgF,flgG,flgH,flgl	Flagellar basal-body rod protein
gtr	gtrA,gtrB	Bactoprenol glucosyl transferase
acp	acpXL	Acyl carrier protein
bcf	bcfA,bcfB,bcfC	Fimbrial subunit
fep	fepA,fepB,fepC,fepD,fepG	Ferrienterobactin transporter
ure	ureB,ureG	Urease protein

### Effects of *Enterobacter cloacae* on the crystal deposition and apoptosis

**Fig. 7:**
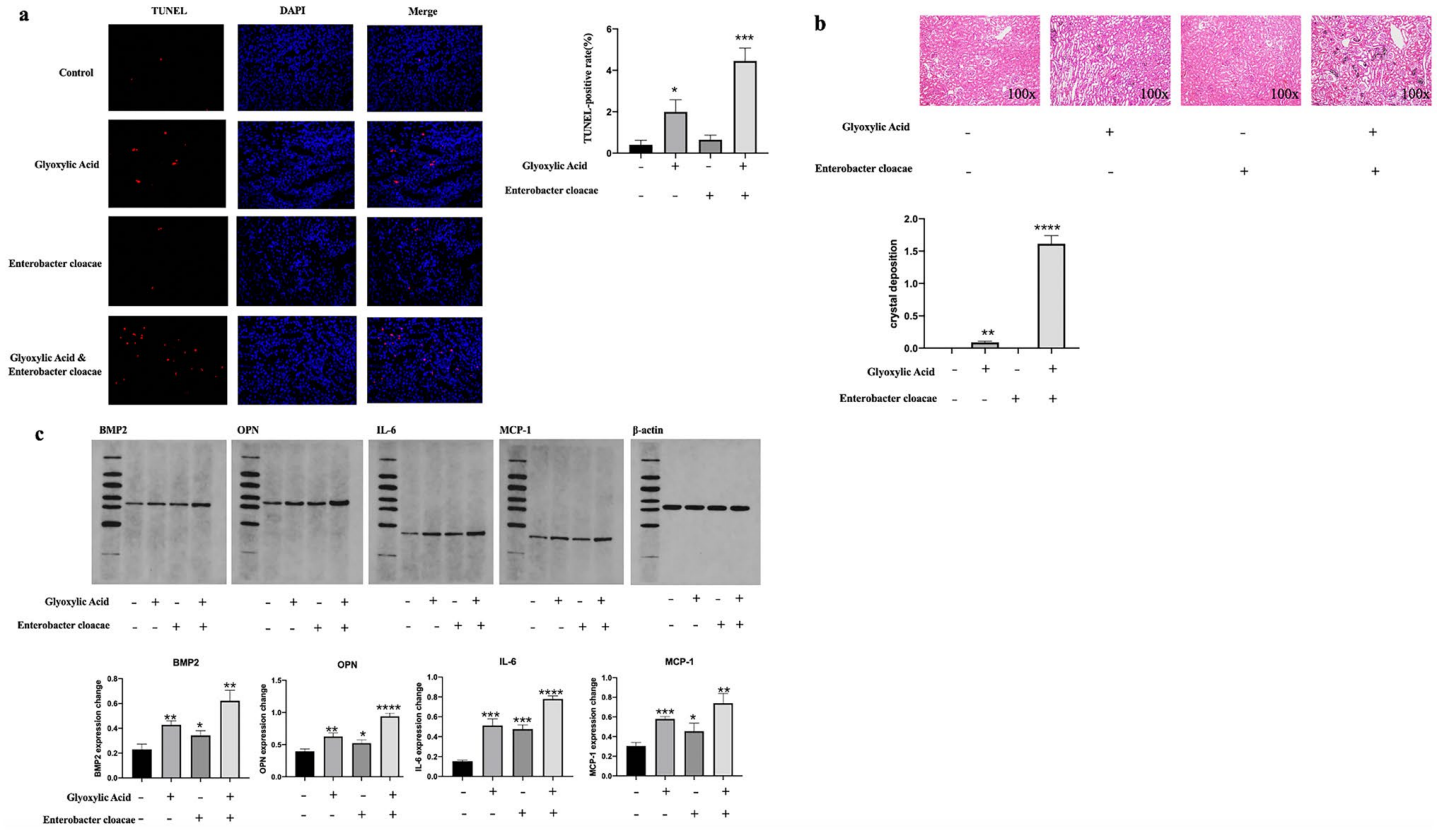
Effects of *Enterobacter cloacae* on the pathophysiological changes in the renal of SD rats. **a** Representative micrographs showed TUNEL staining images of different groups from indicated rats to assess apoptosis. The data are expressed as mean ± SE. *P < 0.05 compared with the control group. **b** Von Kossa staining to detect crystal deposition in the rat kidneys, Glyoxylic Acid and *Enterobacter cloacae* aggravated the crystal depositions compared with other groups. The data are expressed as mean ± SE.*P < 0.05 compared with the control group. **c** Western blot assay showed that the expression levels of BMP2, OPN, IL-6, MCP-1 increased in the kidney tissues in the Glyoxylic Acid or *Enterobacter cloacae* groups compared with those in the control group. Glyoxylic Acid and *Enterobacter cloacae* together could apparently enhance these effects. Western blot bands were quantified using ImageJ software. The data are expressed as mean ± SE. *P < 0.05 compared with the Control group

Apoptosis was assessed using Terminal Deoxynucleotidyl Transferase dUTP Nick-End Labeling (TUNEL) assays. Rats injected Glyoxylic Acid with pre-injection of *Enterobacter cloacae* into renal pelvis expressed severer apoptosis than rats only injected Glyoxylic Acid or *Enterobacter cloacae* (Fig. 7a). Furthermore, Crystal depositions were examined using von Kossa staining. Crystal depositions of rats injected Glyoxylic Acid with pre-injection of *Enterobacter cloacae* into renal pelvis were apparently severer than rats only injected Glyoxylic Acid or *Enterobacter cloacae* (Fig. 7b).

### Effects of *Enterobacter cloacae* on the expression of BMP2, OPN, IL-6 and MCP-1

Western blot analysis indicated that the expression of inflammation-associated proteins IL-6, MCP-1 and osteoblast-associated protein BMP2 and osteopontin (OPN) notably increased in rats injected Glyoxylic or *Enterobacter cloacae* compared with that in control rats, and these increases were significantly enhanced when these rats had been treated with both *Enterobacter cloacae* and Glyoxylic Acid (Fig. 7c).

## Discussion

The components of kidney stones can be classified into following types: calcium oxalate, calcium phosphate, struvite, purine, or cystine. Infection has long been considered to be only associated with struvite, most bacteria present in these stones, such as *Proteus* species, *Klebsiella*, *Pseudomonas* and *Corynebacterium* species, promote the formation of struvite stones via urease [10]. However, struvite stones account for only 4% of all urinary stones [23]. Bacteria have shown to be more broadly associated with kidney stones, patients with kidney stones often present with concomitant urinary tract infection, regardless of stone composition [24]. The majority of urinary stones are calcium oxalate combined with apatite. Previous studies of CaOx stones used to focus on metabolic and genetic factors. However, kidney stones are often unilateral which cannot be explained by such systematic factors. There may exist some local factors contributing to stones formation. What’s more, some urease-negative organisms, for example, *Escherichia coli*, have been found in calcium-based stones. Therefore, bacteria may be associated with CaOx stones [14]. Though many studies have revealed the association, the role bacteria playing in CaOx stones formation remains poorly understood.

Many studies have explored the microbiome of calcium-based urinary stones. Barr-Beare E and his colleague found the dominant bacterial communities of CaOx stones including Enterobacteriaceae and the genera *Pseudomonas* [25]. Ryan et al. added to these bacterial communities with additional identification of *Staphylococcus*, *Veillonella*, *Streptococcus*, *Corynebacterium*, *Haemophilus*, *Lactobacillus*, and *Bifidobacterium* [14]*.* However, there are still many limitations in previous studies. Most studies collected stones or bladder urine though patients only have one-side stones, microbiota of stones or bladder urine are a mixture of two sides upper urinary tracts which cannot represent the difference between the stone sides and non-stone sides kidney. To replenish the gap, we collected pelvis urine of both stone sides and non-stone sides of patients with unilateral CaOx stones to make self-control. Thus, we can diminish the influence of systemic factors and explore the local factors which relate to CaOx stones. Moreover, patients’ antibiotic exposures prior to 4 weeks were not tracked in case antibiotics destroy the bacteria initially colonized [26]. Next-generation 16S rRNA gene sequencing was conducted to the pelvis urine collected from both sides of patients’ kidney, the results can be accurate to the species level; thus, we can have a clear cognition of the difference between stone sides and non-stone sides kidneys.

Through the 16S rRNA gene sequencing of urine samples, we found there were significant differences in 26 species of bacteria in bilateral pelvis urine, these bacteria were enriched in PWY-6478, PWY-6572, PWY-7374, PWY-6944 (MetaCyc database), the bacteria enriched in stone sides pelvis urine were mostly enriched in PWY-6944 pathway (androstenedione degradation). Androstenedione is an androgen, urinary androgens are significantly associated with urinary calcium and citrate excretion, which will finally lead to stones formation [7]. What’s more, among the 26 differentially expressed bacteria in bilateral pelvis urine, *Enterobacter cloacae* and *Chryseobacterium* sp. were most enriched on the stone sides’ pelvis urine. *Chryseobacterium* sp. is a phosphate-solubilizing bacteria (PBS), it was often isolated from soil [27]. At present, the studies on it mostly focus on agriculture. What’s more, Ryan A. Dornbier and his colleagues have found that *Enterobacter cloacae* was isolated from CaOx stones with concordant enrichment on 16S rRNA gene sequencing. Thus, we did not pay much attention to *chryseobacterium*. Meanwhile, the exploration of *Enterobacter cloacae* has increased yearly. *Enterobacter cloacae* is important symbiotic bacteria with intestine, belonging to *Enterobacter* genus, Enterobacter family, Enterobacter order. It usually exists in the intestinal tract of 40–80% human body, and can also exist in the respiratory and urinary tract, causing urinary and respiratory tract infections [28]. *Enterobacter cloacae* has strong adhesion and weak invasion, and can play a role by rearranging cytoskeleton proteins into host cells [28, 29], which are mainly involved in the regulation of pathological processes, such as inflammatory response, oxidative stress and lipid metabolism of host cells [30]. In addition, studies have found that *Enterobacter cloacae* can activate inflammatory and autophagy pathways, triggering metabolic disorders in the body, resulting in heterotopic calcification, namely the formation of atherosclerotic plaques [31]. These suggest that the formation of Randall’s calcium plaques as ectopic calcification of the kidney may also be regulated by *Enterobacter cloacae*. To the best of our understanding, we are the first to discover *Enterobacter cloacae* existing in pelvis urine of patients with CaOx stones. The mechanism of *Enterobacter cloacae* inducing CaOx stones still remains unclear.

To further investigate the role *Enterobacter cloacae* plays in CaOx stones formation, we conducted Whole Genome Sequencing of *Enterobacter cloacae*. Bacteria act on the receptor cells through their virulence factors, or their metabolites, extracellular vesicles. Virulence factors are components of bacterial virulence, and they act in two forms: invasiveness and toxin. By comparing the gene coding protein sequence of the *Enterobacter cloacae* with the amino acid sequence in the VFDB (Virulence Factors of Pathogenic Bacteria) database, we found that flagellum, lipopolysaccharide, aeromycin and urease had high homology, among which flagellum-related protein abundance was the highest. Flagellin is an important scaffold of bacterial movement, dynamic element and virulence factor. Flagellin is the only specific ligand of Toll-like receptor 5, the host membrane protein receptor, which has strong immunogenicity. As a unique structural protein of bacteria, flagellin is also one of the most abundant proteins in bacteria. As a virulence factor, flagellin can promote the invasion of pathogenic bacteria. Therefore, flagellin is a key target for host immune monitoring. When pathogenic bacteria invade the host, the natural immune receptor TLR5 can easily recognize flagellin, thus inducing MAPK- and NF⁃κB-mediated inflammatory response [32]. Some investigators have reported that Flagellin could accelerate CaOx deposits by promoting its crystallization, growth and aggregation [12]. What’s more, flagellin could also facilitate Ca^2+^ influx via calcium cellular membrane channel [33]. However, the association of flagellin with CaOx deposits has been only superficially studied, more studies should be applied to explore the mechanism of flagellin inducing CaOx deposits.

KEGG and GO analysis revealed *Enterobacter cloacae* may play important roles in ion binding and signaling transduction. Cherng et al. found that bacterial infection contributed to the formation of CaOx deposits via calcium-related ion channels after injected bacteria [34]. Cell signal transduction is a process in which cells experience the stimulation of information molecules through cellular membrane or intracellular receptors, and then transform by intracellular signal transduction system, thus affecting the biological functions of cells. It broadly existed in many biological processes. There are many signaling transduction pathways involved in CaOx stones formation. The P-38/JNK MAPK transduction pathway is turned on in the process of CaOx stone formation [35].

To validate our previous claims, we have conducted in vivo experiments. We assessed the apoptosis condition and crystal depositions of kidneys between rats with different treatments. Pre-injected with *Enterobacter cloacae* could apparently accelerate the apoptosis and crystal depositions of metabolic disorders rats induced by Glyoxylic Acid. While injected Glyoxylic or *Enterobacter cloacae* alone did not make any difference in apoptosis condition and crystal depositions comparing to control groups. What’s more, the expression of IL-6, MCP-1, BMP2 and OPN was higher in rats injected with Glyoxylic Acid or *Enterobacter cloacae* than control. Treated with both *Enterobacter cloacae* and Glyoxylic Acid could enhance the increases of these proteins. It has been previously demonstrated that IL-6 and MCP-1 indicated a close relationship between inflammation and kidney stones, while our previous research has confirmed that osteogenic transformation-related proteins BMP2 and OPN played important roles in stone formation [36, 37]. Thus, we could presume that pre-existence of *Enterobacter cloacae* will enhance the effects of metabolic disorders in CaOx stones formation through inflammation process and osteogenic transformation. However, the process of CaOx stones formation is complex, bacteria themselves might cause infection that induce inflammatory response and macrophages recruitment [38]. We cannot be sure that *Enterobacter cloacae* is the special regulator of CaOx stones formation via inflammation and osteogenic transformation, or if this is a general phenomenon with kidney infection. But our study is just a preliminary exploration based on a finding that there exist different microbiota in stone sides and non-stone sides pelvis urine of patients with unilateral CaOx stones and our in vivo experiments demonstrates that *Enterobacter cloacae* has associations with CaOx stones formation, while the specific mechanism still needs to be further explored. In addition, we look forward to future studies which can explore the relationship of other bacteria with stone formation.

In summary, our results revealed that the occurrence of bacteria is considerably correlated with CaOx stones formation. *Enterobacter cloacae* and its flagellin are supposed to have effects on CaOx stone formation through ion binding and signaling transduction. In vivo experiments we conducted have identified that *Enterobacter cloacae* could accelerate the stone formation of rats with metabolic disorders. However, we just hypothesized the possible process of CaOx stone formation; further studies are required to explore the detailed mechanism of *Enterobacter cloacae* and its Flagellin inducing CaOx stone formation. What’s more, more patients should be recruited in our studies to increase the trustworthiness of our outcomes.

## Conclusion

Microbiome colonized in kidney are associated with kidney stone formation. *Enterobacter cloacae* colonized in kidney can accelerate CaOx stone formation, while the concrete mechanism still needs more explorations.

## Supplementary Information

Below is the link to the electronic supplementary material.

Supplementary file1 (JPG 2259 KB)

Supplementary file2 (JPG 595 KB)

Supplementary file3 (JPG 603 KB)
