# Supplementary figures and images for: Enterobacter cloacae: a villain in CaOx stone disease?
Source: Urolithiasis. 2022 Feb 6;50(2):177–88. doi: 10.1007/s00240-022-01311-8 (PMC8956555; doi:10.1007/s00240-022-01311-8)

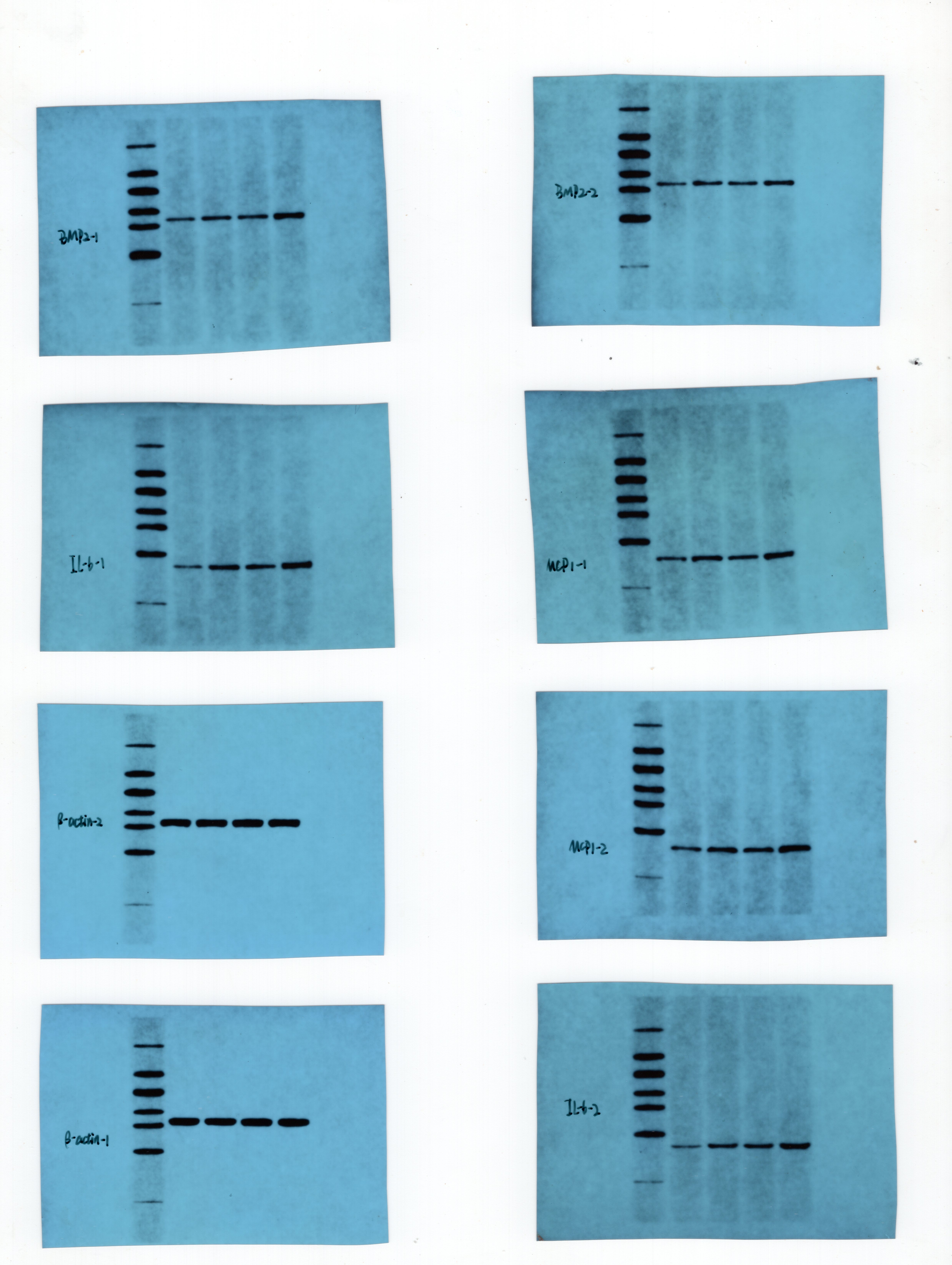

Supplement: Supplementary file 1 — Supplementary file1 (JPG 2259 KB) [file 240_2022_1311_MOESM1_ESM.jpg]

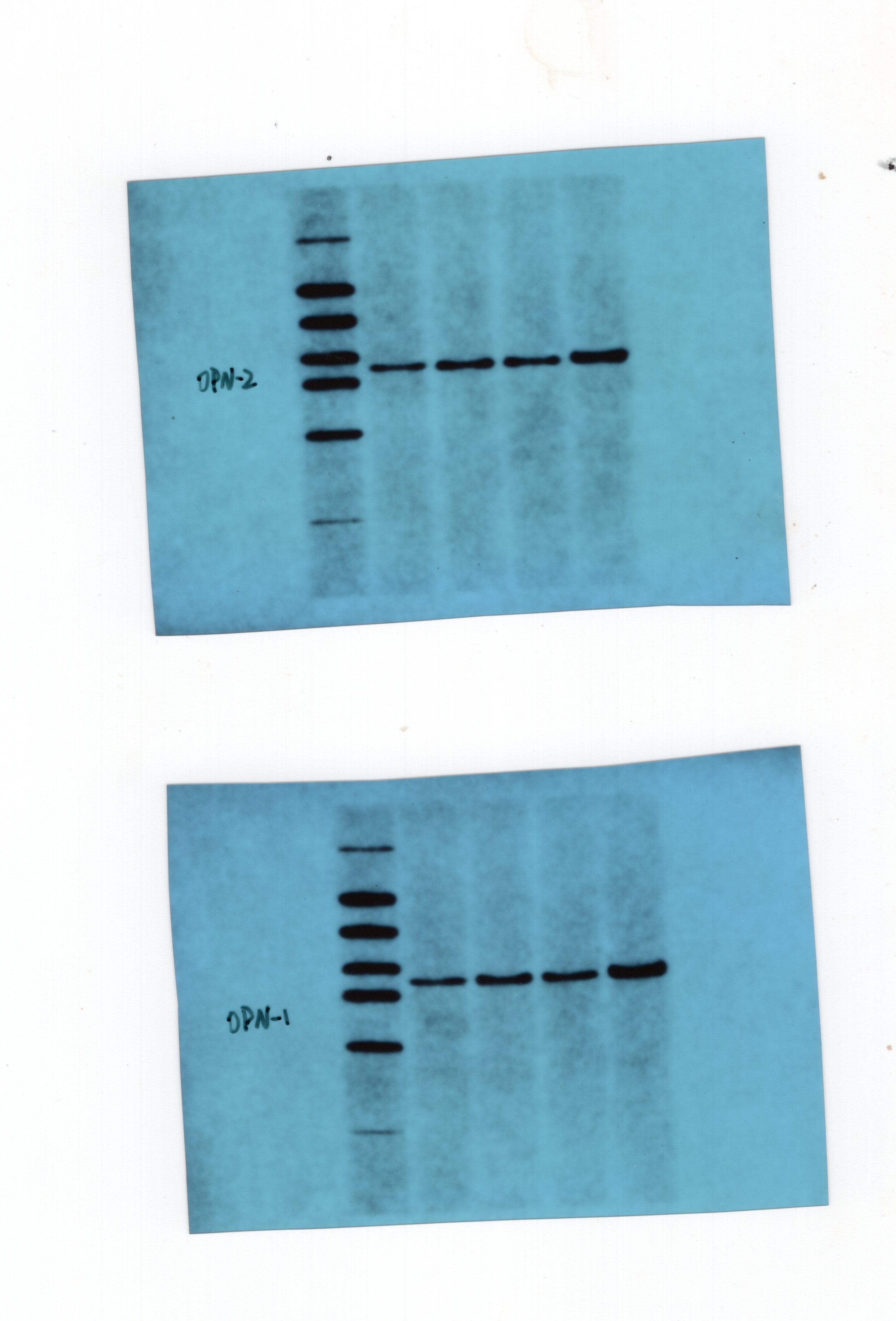

Supplement: Supplementary file 2 — Supplementary file2 (JPG 595 KB) [file 240_2022_1311_MOESM2_ESM.jpg]

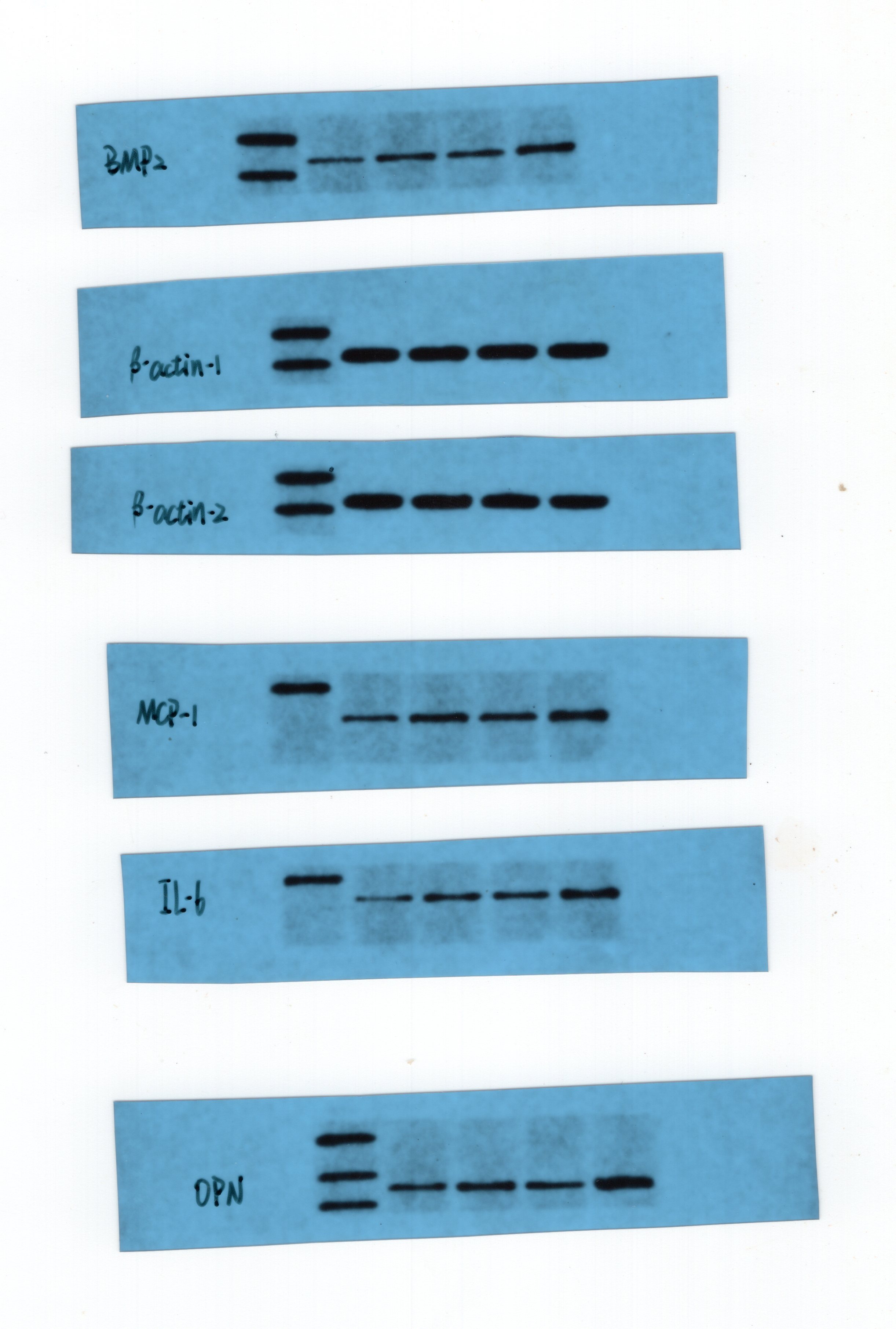

Supplement: Supplementary file 3 — Supplementary file3 (JPG 603 KB) [file 240_2022_1311_MOESM3_ESM.jpg]
